# A 78-year-old female with severe tongue pain: benefit of modern ultrasound

**DOI:** 10.1186/s12880-021-00585-5

**Published:** 2021-03-20

**Authors:** Lara Clarissa Burg, Wolfgang Andreas Schmidt, Peter Brossart, Katharina Isabell Reinking, Franziskus Maria Schützeichel, Robert Patrick Finger, Valentin Sebastian Schäfer

**Affiliations:** 1 Clinic of Internal Medicine III, Department of Oncology, Haematology, Rheumatology and Clinical Immunology, University Hospital Bonn, Venusberg-Campus 1, 53127 Bonn, Germany; 2 Medical Center for Rheumatology Berlin-Buch, Immanuel Krankenhaus Berlin Buch, Berlin, Germany; 3 Department of Ophthalmology, University Hospital Bonn, Bonn, Germany

**Keywords:** Giant cell arteritis, Vasculitis, Ultrasound, Follow-up, Intima-media thickness, Lingual artery, Central retinal artery

## Abstract

**Background:**

Giant cell arteritis (GCA) is the most common form of systemic vasculitis in persons aged 50 years and older. Medium and large vessels, like the temporal and axillary arteries, are commonly affected. Typical symptoms are headache, scalp tenderness, jaw claudication and ophthalmological symptoms as loss of visual field, diplopia or amaurosis due to optic nerve ischemia. Tongue pain due to vasculitic affection of the deep lingual artery can occur and has so far not been visualized and followed up by modern ultrasound.

**Case presentation:**

We report the case of a 78-year-old woman with typical symptoms of GCA, such as scalp tenderness, jaw claudication and loss of visual field, as well as severe tongue pain. Broad vasculitic affection of the extracranial arteries, vasculitis of the central retinal artery and the deep lingual artery could be visualized by ultrasound. Further did we observe a relevant decrease of intima-media thickness (IMT) values of all arteries assessed by ultrasound during follow-up. Especially the left common superficial temporal artery showed a relevant decrease of IMT from 0.49 mm at time of diagnosis to 0.23 mm on 6-months follow-up. This is the first GCA case described in literature, in which vasculitis of the central retinal artery and the lingual artery could be visualized at diagnosis and during follow-up using high-resolution ultrasound.

**Conclusion:**

High-resolution ultrasound can be a useful diagnostic imaging modality in diagnosis and follow-up of GCA, even in small arteries like the lingual artery or central retinal artery. Ultrasound of the central retinal artery could be an important imaging tool in identifying suspected vasculitic affection of the central retinal artery.

## Background

Giant-cell arteritis (GCA) is the most common form of primary systemic vasculitis in persons aged 50 years and older [1].

The clinical presentation of GCA is variable, but common symptoms are headache, hypersensitivity of the scalp, jaw claudication and visual symptoms.

Affection of the tongue is rare but can result in tongue necrosis [2] and may be misdiagnosed as temporomandibular dysfunction [3].

We report a case of an elderly female with GCA with considerable affection of extracranial arteries including the lingual and the central retinal arteries. These could be visualized and followed up with high-resolution ultrasound. Based on our findings, ultrasound is a very useful adjunct diagnostic and monitoring imaging modality.

## Case presentation

A 78-year-old female was referred as an emergency to the ophthalmology department of the University Hospital Bonn. She reported blurred vision for seven days and loss of visual field as well as photopsia for 2 days. Amaurosis or diplopia were not reported.

She had dry cough for 4 months. This was refractory to symptomatic and antibiotic therapy.

In the past 5 weeks, the patient reported increasing diffuse thrusting, bilateral pain in the area of the mandible, while opening her mouth and while chewing. In addition, she noted a thrusting pain in her tongue, which initially increased postprandially but soon was present continuously, and led to difficulties in swallowing. The patient also reported weight loss of 4 kg over the past 4 weeks, increasing fatigue and subfebrile body temperatures as well as scalp tenderness. Severe headaches of the temporal area were denied, as well as myalgia and morning stiffness.

All symptoms started on the left side, but affected both sides over time.

Examinations by a dentist, an orthodontist and an otolaryngologist remained without any findings.

The patient was alert, orientated and had appropriate affect on clinical examination.

The neurological examination remained unremarkable, without dolorous nerve exit points and a negative swinging flashlight test. The tongue appeared normal, with no diversion, swelling or necrosis. Palpation of the temporal arteries was normal.

Chest radiography was without pathologies.

The patient reported neither a history of vasoconstrictive medication, nor was there a history of radiotherapy involving any site of the body.

Laboratory results revealed increased inflammatory markers. C-reactive protein (CRP) was 71 mg/l (reference range, 0 to 5 mg/l) and erythrocyte sedimentation rate was 75 mm/h (reference range, < 30 mm/h).

**Fig. 1:**
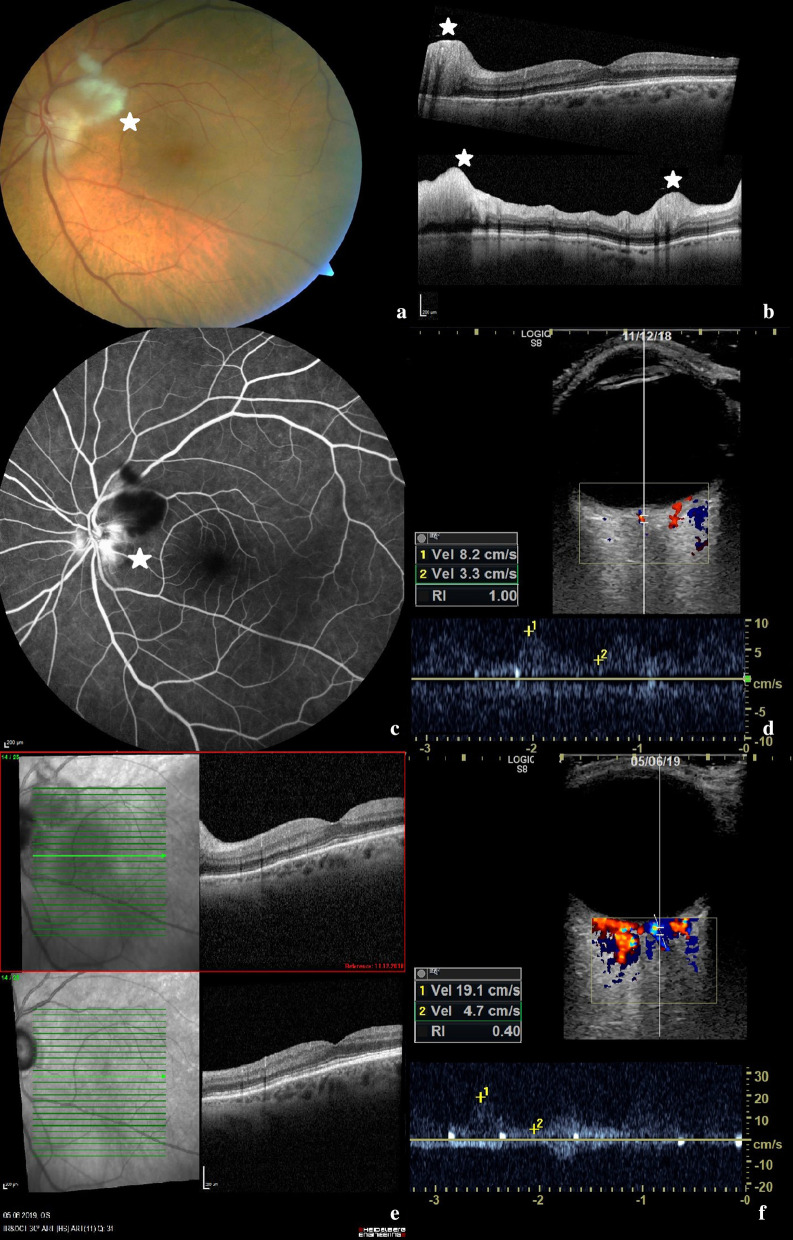
Ophthalmologic imaging and transocular ultrasound of the left eye. **a** Colour fundus retinal photography of the left eye displaying an optic disc edema with adjacent cotton wool spot (*) along the upper temporal vascular arch. **b** Macula-optical coherence tomography of the left eye with a normal macula and segmental disc edema. Retinal nerve fibre layer thickness was increased in the superior, temporal and inferior segments (*). **c** Fluorescein angiography of the left eye with distinct hyperfluorescence of the papilla, i.e. a “hot disc”. **d** Transocular ultrasound including measurement of flow velocity of the central retinal artery of the left eye on admission. **e** Macula-optical coherence tomography on admission (top) compared to 6-month follow-up (bottom). **f** Transocular ultrasound including measurement of flow velocity of the central retinal artery of the left eye in 6-month follow-up

On admission, the best corrected visual acuity was 20/400 in the left and 20/25 in the right eye. Funduscopic examination of the left eye revealed optic disc edema with cotton wool spots along the upper temporal vascular arch. The macula and the retinal blood vessels appeared normal on fundoscopy (Fig. 1a).

Optical coherence tomography (OCT) of the left macula was normal and OCT of the optic disc confirmed disc edema in accordance with the clinical appearance. Retinal nerve fibre layer thickness was increased in the superior, temporal and inferior segments (Fig. 1b) and the optic disc showed diffuse leakage (“hot disc”) on fluorescein angiography (Fig. 1c). Based on these ophthalmological findings, anterior ischemic optical neuropathy (AION) of the left eye was diagnosed.

Following this, the patient was referred to the Department of Rheumatology at the University Hospital Bonn for further evaluation. The patient underwent ultrasound examination of all arteries typically involved in GCA according to OMERACT protocol [4]. A GE Logiq S8 XDclear ultrasound machine with software version R3 manufactured in 2018 was used. For sonographic examination of the axillary, vertebral and carotid arteries, a linear ultrasound probe with a range from 6 to 15 MHz was used, for all other small arteries including the central retinal artery an ultrasound probe with a range from 8 to 18 MHz was applied.

**Fig. 2:**
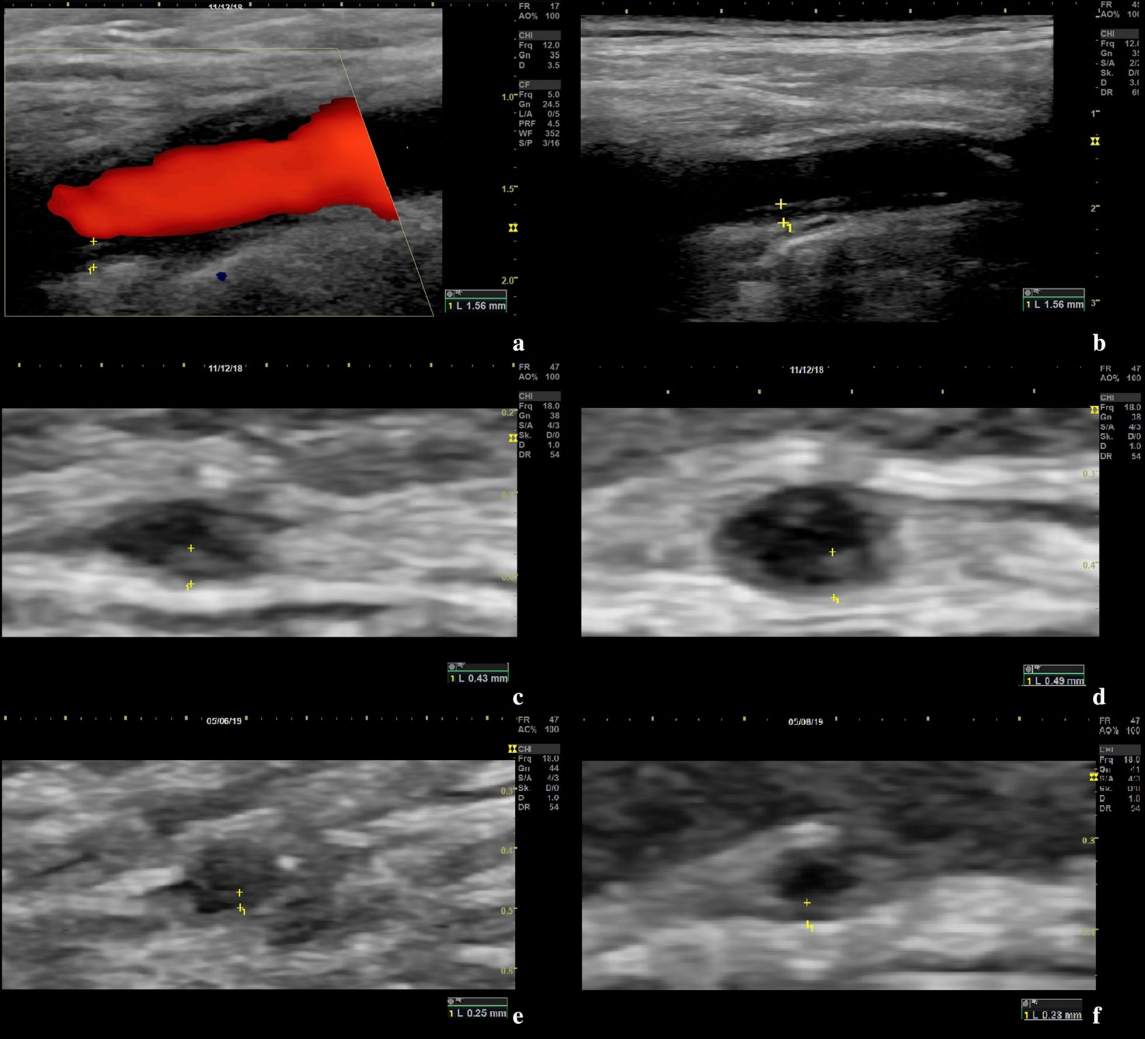
Ultrasound images displaying vasculitic changes of the intima-media complex of the respective arteries. **a**, **b** Left vertebral artery in Doppler and B-mode ultrasound with pathological vessel wall and increased intima-media thickness (IMT) due to inflammation. **c** Right common superficial temporal artery with pathological IMT of 0.43 mm. **d** Left common superficial temporal artery with pathological IMT of 0.49 mm. **e** Right common superficial temporal artery in 6-month follow-up with IMT of 0.25 mm. **f** Left common superficial temporal artery in 6-month follow-up with IMT of 0.23 mm

Vascular ultrasound demonstrated a homogeneous, concentric thickening of the intima-media complex, known as halo sign, in several arteries of the head and neck area (Fig. 2) [5, 6]. Ultrasound was performed on axillary arteries, vertebral arteries, common carotid arteries, superficial temporal arteries with both frontal and parietal branches, occipital arteries, facial arteries, and due to the lingual pain, also the lingual artery. Vasculitic affection with increased intima-media thickness (IMT) values above published cut-off values was observed in the common superficial temporal arteries on both sides, both frontal and parietal branches (up to 0.49 mm), as well as in the right facial artery (0.59 mm) and right axillary artery (1.09 mm) [7]. A halo sign was also visible in both vertebral arteries. Exact values and the respective cut-off values are depicted in Table 1.

**Table 1:** Intima-media thickness values on admission, at 3-month and 6-month follow-up

Artery	IMT cut-off value (mm) [7]	Right side (mm)			Left side (mm)		
		Admission	After 3 months	After 6 months	Admission	After 3 months	After 6 months
Axillary artery	1.0	1.09*	0.73	0.62	0.73	0.60	0.63
Vertebral artery	not defined	1.96	0.45	0.57	1.56	0.58	0.43
Common superficial temporal artery	0.42	0.43*	0.25	0.25	0.49*	0.30	0.23
Frontal branch	0.34	0.39*	0.33	0.19	0.37*	0.26	0.15
Parietal branch	0.29	0.44*	0.30*	0.19	0.49*	0.33*	0.17
Occipital artery	not defined	0.28	0.23	0.15	0.22	0.20	0.14
Facial artery	0.37	0.59*	0.23	0.23	0.25	0.34	0.23

*IMT* Intima-media thickness, pathological values marked with *

**Fig. 3:**
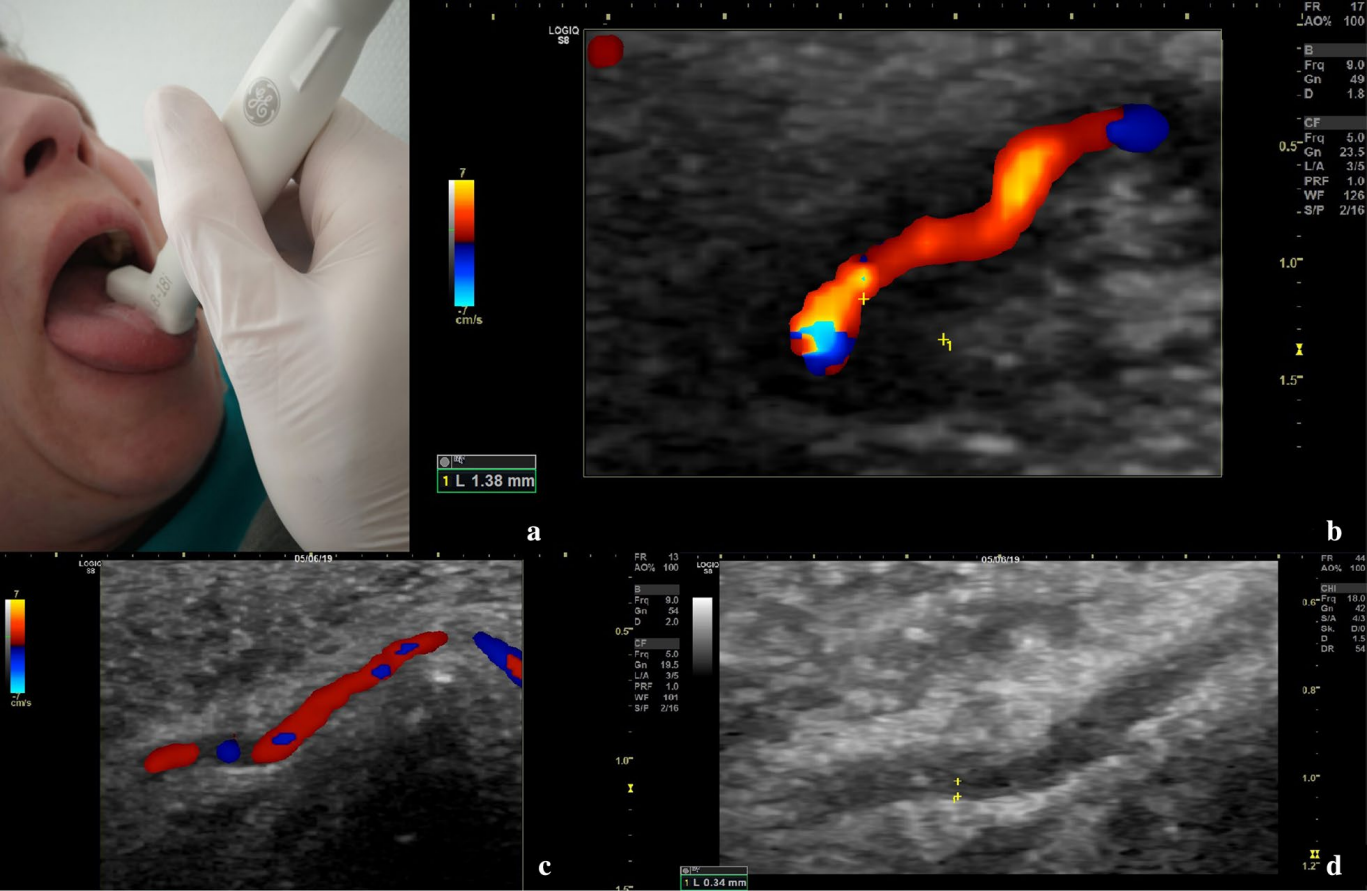
Ultrasound examination of the lingual artery with an 18 MHz hockey stick probe. **a** Position of the ultrasound probe on the patient´s tongue. **b** Doppler ultrasound and intima-media thickness (IMT) values of the affected deep lingual artery. **c** Doppler ultrasound of the deep lingual artery on 6-month follow-up, reduced field of view. **d** IMT values of the deep lingual artery on 6-month follow-up in B-Mode, reduced field of view

Furthermore, due to tongue claudication and lingual pain, the deep lingual artery, which is a branch of the lingual artery, branching from the external carotid artery, was also examined by ultrasound. It displayed a typical vasculitic halo-sign (Fig. 3). Vasculitic IMT swelling led to markedly reduced blood flow and visible IMT thickening in B-mode. Peak IMT of the lingual artery was 1.38 mm. There was no occlusion of the lingual artery. This sonographic finding explained the patient´s symptoms of thrusting pain of her tongue.

In addition, transocular ultrasound of the central retinal artery was performed on both sides. Retinal artery displayed a systolic flow velocity of 8.2 cm/s on the left eye, and 7.5 cm/s on the right eye. This meant a marked reduction on both eyes, including the asymptomatic right eye. Until now, we have examined 25 healthy individuals with a mean systolic velocity of 14.4 cm/s (SD ± 3.2) of the central retinal artery.

The ocular ultrasound findings were in agreement with both the patient’s visual symptoms and the ophthalmological findings mentioned above.

Diagnosis of GCA was made based on the patient´s symptoms, clinical examination, laboratory results and ultrasound findings, following the EULAR recommendations for imaging in GCA [8]. Therefore, therapy was promptly initiated. Due to the characteristical sonographic findings no biopsy of the superficial temporal artery or any other artery was performed.

The patient received an initial daily dose of 500 mg methylprednisolone intravenous for five days followed by a daily dose of 60 mg prednisolone per os, tapered over 26 weeks according to the GIACTA treatment protocol [9]. Furthermore, tocilizumab treatment was initiated with a weekly dose of 162 mg subcutaneously due to the widespread affection of the arterial vascular bed.

With treatment symptoms and laboratory markers quickly improved. Jaw claudication and tongue pain resolved within days and did not reoccur. The patient was dismissed five days later in stable clinical condition.

Three months after dismissal a follow-up examination was performed. The patient was on 9 mg prednisolone per day, 162 mg tocilizumab subcutaneously per week and free of symptoms. CRP was normal. On ultrasound examination a relevant decrease of IMT values could be observed in all affected arteries. IMT of the deep lingual artery had decreased from 1.38 to 0.77 mm. Systolic flow velocities of both central retinal arteries had increased from 8.2 to 21.6 cm/s on the left eye and from 7.5 to 19.1 cm/s on the right eye.

At ophthalmological and sonographic follow-up examination 6 months after the patient´s dismissal, best corrected visual acuity increased from 20/400 to 20/40 in the left eye, the right eye was still without pathological finding. Funduscopic examination of the left eye showed a decrease of optic disc edema with a pale optic disc and narrow vessels.

OCT of the left macula revealed atrophy of inner retinal layers (Fig. 1e), while OCT of the optic disc confirmed absence of disc edema and a decrease of retinal nerve fibre layer thickness.

The patient was on 12.5 mg prednisolone per day and 162 mg tocilizumab subcutaneously per week and free of symptoms. The dose of prednisolone was increased for a short time by an external rheumatologist, due to recurring headaches and CRP-elevation between the follow-up examinations.

On ultrasound examination, a further decrease in IMT values could be observed (Table 1). Flow velocity of both central retinal arteries showed stable values with 19.1 cm/s on the left eye and 17.2 cm/s on the right eye. IMT of the deep lingual artery had decreased from 0.77 to 0.34 mm.

Therefore, tapering of prednisolone and therapy with tocilizumab was continued following the GIACTA protocol [9].

## Discussion and conclusion

This case illustrates a rare complication of GCA—vasculitis of the lingual artery, which could be diagnosed and followed-up by modern ultrasound. Clinical presentation of vasculitic affection of the tongue without respective imaging is described only in a few cases in literature [10, 11]. Tongue necrosis is described as a rare but severe complication [2, 12].

This is the first case described in the literature, in which vasculitis of the lingual artery could be assessed and documented by ultrasound.

Further is this the first case in literature in which vasculitic vessel wall edema of the central retinal artery was visualized, which markedly decreased over 3 and 6 months under therapy. The patient´s symptoms rapidly disappeared including the dry cough which has been described in 14% of GCA patients and often occurs before other symptoms appear [13, 14].

As highlighted by the improved systolic flow velocity of the central retinal artery on follow-up, ischemia of the optic nerve was likely not permanent and improved under treatment, which might explain the good visual recovery. To this point there is no literature examining the ultrasound measurement of the flow velocities of the central retinal artery, but there seems to be a significant decrease in flow velocities, as recently published at the EULAR congress in 2020 [15].

In conclusion, this case illustrates the importance of a fast ultrasound examination of the commonly affected arteries in suspected GCA. Diagnostic imaging should not delay treatment with glucocorticoids, especially in the case of specific visual symptoms. This report raises the question, whether the use of ultrasound is also useful in examining arteries smaller than the lingual artery. As highlighted above, it was possible to measure flow velocities of the central retinal artery, which is a smaller medium size vessel. To this point it remains uncertain, to which extent smaller medium-sized arteries (e.g. the posterior auricular artery) can reliably be assessed by ultrasound. A current prospective study performed by this group is addressing this question.

The role of ultrasound in diagnosis of GCA is known [8]. Apparently, there also seems to be a role in monitoring disease during follow-up examinations. The patient´s follow-up examinations showed a relevant decrease of IMT values over a 6 months period. Nearly all IMT values of examined arteries decreased below the respective cut-off values (Table 1) [7]. IMT values of the lingual artery noticeably decreased from 1.38 mm at diagnosis to 0.34 mm at 6-months follow-up. Further research regarding the role of ultrasound in follow-up is required. In summary, ultrasound can be a helpful and quickly available imaging modality for diagnosis and follow-up of GCA. Nevertheless, an experienced examiner is required to achieve reliable results [4]. Monti et al. recently published an article assessing the role of ultrasound in GCA by reviewing the available literature including recommendations to improve diagnosis of GCA [16].

In this case report, we furthermore were able to visualize vasculitis of the lingual artery and the central retinal artery with modern high frequency ultrasound probes. Sonographic examination of the central retinal artery can be helpful in understanding vasculitic affection of the eyes at time of diagnosis and in follow-up. Further research is warranted on this subject.

## Data Availability

All data generated and analysed for this case report are included in this published article.

## Abbreviations

AION: Anterior ischemic optical neuropathy
CRP: C-reactive protein
GCA: Giant cell arteritis
IMT: Intima-media thickness
OCT: Optical coherence tomography
